# Nutritional Behavior of Patients with Bone Diseases: A Cross-Sectional Study from Austria

**DOI:** 10.3390/nu16121920

**Published:** 2024-06-18

**Authors:** Daniel A. Kraus, Amadea Medibach, Martina Behanova, Annemarie Kocijan, Judith Haschka, Jochen Zwerina, Roland Kocijan

**Affiliations:** 1 Ludwig Boltzmann Institute of Osteology at Hanusch Hospital of OEGK and AUVA Trauma Centre Meidling, 1st Medical Department Hanusch Hospital, 1140 Vienna, Austria; daniel.kraus@osteologie.lbg.ac.at (D.A.K.); amadea.medibach@icloud.com (A.M.); martina.behanova@osteologie.lbg.ac.at (M.B.); judith.haschka@osteologie.lbg.ac.at (J.H.); jochen.zwerina@osteologie.lbg.ac.at (J.Z.); 2 Metabolic Bone Diseases Unit, School of Medicine, Sigmund Freud University Vienna, 1020 Vienna, Austria; 3 Optimal Essen e.U., 1040 Vienna, Austria; kocijan@optimalessen.com; 4 Vienna Bone and Growth Center, 1130 Vienna, Austria

**Keywords:** nutrition, vitamin D, rare bone disease, X-linked hypophosphatemia, osteogenesis imperfecta, hypophosphatasia, osteoporosis

## Abstract

Background: A balanced diet rich in calcium and protein is recommended for bone-healthy people and osteoporosis patients, but it may also be important for rare bone disease (RBD). Little data is available on RBD and diet. Therefore, the aim of this study was to evaluate the nutritional behavior of patients with RBD. Methods: This single-center, cross-sectional, questionnaire-based study assessed the nutritional behavior of RBD patients (X-linked hypophosphatemia (XLH), osteogenesis imperfecta (OI), hypophosphatasia (HPP)), osteoporosis (OPO) patients and healthy controls (CTRL). The nutritional questionnaire comprised 25 questions from seven nutritional areas. The associations between socioeconomic factors and BMI were assessed by age-adjusted univariate analysis of covariance (ANCOVA). Results: Fifty patients with RBD (17 OI, 17 HPP, 16 XLH; mean age of 48.8 ± 15.9, 26.0% male, mean BMI 26.2 ± 5.6), 51 with OPO (mean age 66.6 ± 10.0, 9.8% male, mean BMI 24.2 ± 3.9) and 52 CTRL (mean age 50.8 ± 16.3, 26.9% male, mean BMI 26.4 ± 4.7) participated. Twenty-six (52.0%) RBD, 17 (33.4%) OPO and 24 (46.1%) CTRL were overweight or obese according to BMI. Only a minority of RBD, OPO and CTRL had a daily intake of at least three portions of milk or milk products (17.3% RBD, 15.6% OPO, 11.6% CTRL, *p* = 0.453). In general, similar nutritional behavior was observed between the three subgroups. However, significant differences were found in caffeine consumption (*p* = 0.016), fruit/vegetable juice consumption (*p* = 0.034), portions of fish per week (*p* = 0.044), high-fat meals per week (*p* = 0.015) and consumption of salty snacks (*p* = 0.001). Conclusion: Nutritional counseling, controlling BMI and ensuring sufficient calcium and protein intake are crucial in patients with osteoporosis as well as in rare bone diseases. Vitamin D does not appear to be sufficiently supplied by the diet, and therefore supplementation should be considered in patients with bone diseases.

## 1. Introduction

Rare bone diseases (RBD), including X-linked hypophosphatemia (XLH), osteogenesis imperfecta (OI) and hypophosphatasia (HPP), are associated with numerous complications such as (pseudo-)fractures, chronic pain and limitations of physical functioning [1,2,3]. These factors, among others, lead to a decreased quality of life in patients with RBD [4]. To date, there is no cure for these diseases, and the available therapeutic options are limited [5,6,7]. These rather poor perspectives force RBD patients to focus more intensely on non-therapeutic options, increasing their mental and physical well-being and preventing disease progression. One major factor is lifestyle, including diet. As nutritional status plays an important role in other bone diseases like osteoporosis, with clear recommendations for certain nutrients like calcium and vitamin D, nutrition is also of importance in RBD [8]. Nutritional habits can influence the well-being of affected subjects [9,10,11], especially in XLH, where the conventional therapy consists of phosphate and active vitamin D supplementation, or in patients with OI receiving antiosteoporosis treatment [9,11].

Furthermore, nutritional status is linked to disease progression by an increase in body mass index (BMI)-driven co-morbidities. In particular, RBD patients are at high risk due to decreased physical activity. Restrictive respiratory patterns due to thoracic bone deformations have been reported in OI patients. Moreover, the co-presence of obstructive sleep apnea was higher in obese OI patients [12]. Moreover, overweight was also reported to be associated with vitamin D deficiency in OI patients [13].

Vitamin D plays a major role in calcium homeostasis and bone mineralization. Despite the diverse etiology and pathophysiological mechanisms of XLH, OI and HPP, sufficient intake of vitamin D and calcium seems mandatory. This might be especially true for OI patients receiving anti-resorptive agents such as bisphosphonates or denosumab. Insufficient vitamin D levels in OI patients have been reported previously [14]. Thus, the correction of vitamin D levels was suggested in children with OI to avoid secondary hyperparathyroidism, hypocalcemia and consequently high bone turnover [15]. Vitamin D deficiency is also a common finding in HPP [16]. Vitamin D supplementation might, therefore, have positive effects. However, for calcium, a balanced diet rather than calcium supplementation was recommended to avoid hypercalcemia. The same is true for XLH, suggesting a balanced diet, rather than calcium supplements. We have recently reported on complementary medicine in patients with bone diseases. Interestingly, supplementations were not common in RBD, and only 26% of patients stated a vitamin D supplementation [5].

Although there are clear nutritional recommendations for osteoporosis as well as for bone healthy people, there is little evidence for nutritional recommendations for patients with RBD. This could be related to the fact that, although XLH, OI and HPP are the “most common” RBD, the incidence and prevalence of these diseases is low, e.g., XLH has an incidence of 3.9 per 100,000 live births [9].

The aim of this study was to evaluate the nutritional behavior of patients with RBD (XLH, OI and HPP), osteoporosis and healthy controls in a specialized center for bone diseases.

## 2. Methods

This single-center, cross-sectional, questionnaire-based study was conducted at Hanusch Hospital of OEGK—a hospital affiliated with the Vienna Bone and Growth Center (European Reference Network Center for Rare Bone Disease)—in cooperation with the Ludwig Boltzmann Institute of Osteology. The study population consisted of 3 groups: (i) patients with rare bone diseases (RBD), (ii) osteoporosis patients (OPO) and healthy controls (CTRL). RBD included X-linked hypophosphatemia (XLH), hypophosphatasia (HPP) and Osteogenesis Imperfecta (OI) Sillence type I–IV. RBD and OPO patients were recruited from the outpatient clinic specializing in bone diseases and rare bone diseases at Hanusch Hospital. The CTRL group consisted of volunteers (hospital staff, visitors), patients in need of orthopedic surgery (non-osteoporotic fractures, other injuries, need of endoprosthesis) or other surgical procedures. The inclusion criteria comprised age ≥ 18 years and skills in the German language. Subjects with higher-grade dementia, that did not sign the written consent or fulfill the inclusion criteria were excluded.

**Figure 1:**
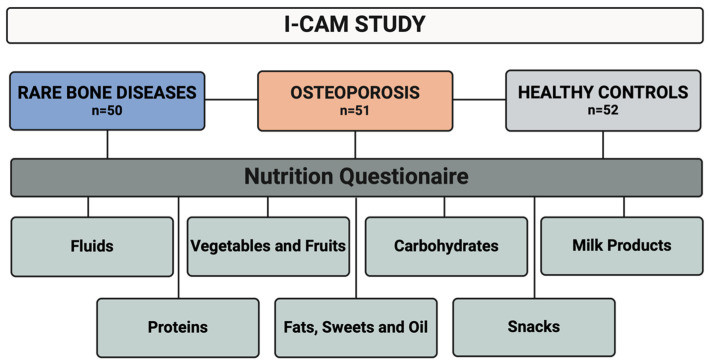
Study flow chart. The nutrition questionnaire comprised 25 questions from 7 nutritional domains: 1. fluids, 2. vegetables and fruits, 3. carbohydrates, 4. milk products, 5. proteins, 6. fat, sweets and oil and 7. snacks.

In this study, we applied a nutritional questionnaire commonly used in the routine clinical practice of German general practitioners (GPs). Prior to its administration, the questionnaire’s relevance and appropriateness for this study were carefully reviewed and confirmed by a panel of experts from the nutritional and bone fields. This ensured that the tool was well-suited to the clinical contexts it aimed to investigate, maintaining the practical applicability of the findings. The questionnaire comprised 25 questions in German regarding nutritional behavior as well as demographic aspects. The questionnaire consisted of seven nutritional categories: 1. fluid, 2. vegetables and fruits, 3. carbohydrates, 4. milk products, 5. proteins, 6. fat, sweets and oil and 7. snacks. It covered all aspects of nutrition relevant to bone health. The questions were mainly designed as single-choice questions with multiple answers available. Fill-out time was assumed to be approximately 10 min. In case of difficulties filling out the questionnaire, staff was available for guidance. Furthermore, additional demographic information was obtained along with the questionnaire. The study flow chart is displayed in Figure 1, the entire questionnaire is in Supplementary Table S1.

For data protection purposes, all subjects were pseudonymized. Only authorized personnel of Hanusch Hospital and the Ludwig Boltzmann Institute of Osteology had access to the data. The study was approved by the local ethics committee (ethics committee of the city of Vienna; protocol number: EK 20-214-VK; date of approval: 10 November 2020), reported to the hospital management of the Hanusch Hospital, and carried out in accordance with the Declaration of Helsinki.

### Statistics

Data concerning age, sex, marital status, highest educational attainment and employment status were obtained through a questionnaire. Educational attainment was categorized as basic (including primary education), secondary (comprising high school with and without completion of the leaving examination) and tertiary (encompassing university education). Educational level, marital status (single, married or cohabiting, divorced or widowed) and employment status (employed or unemployed) were utilized as indicators of socioeconomic status. Body mass index (BMI) was calculated from the self-reported height and weight by dividing a patient’s weight in kilograms by the square of their height in meters. BMI was then categorized according to the World Health Organization (WHO) guidelines: underweight (BMI < 18.5), normal weight (BMI 18.5–24.9), overweight (BMI 25.0–29.9) and obese (BMI ≥ 30). To describe the characteristics of the RBD, OPO and CTRL groups, frequencies and percentages were used for categorical variables. For continuous variables, the decision to use means and standard deviations (SD) or medians and interquartile ranges (IQR) depended on assessments of normality using the Shapiro–Wilk test.

Differences in patient groups regarding demographic parameters and nutritional behaviors were assessed using the Pearson chi-square test for categorical variables and the independent-samples Kruskal–Wallis test for continuous variables, after verifying the assumption of non-normal distribution for the latter.

To explore associations between selected socioeconomic factors (educational level, employment status and marital status) and BMI across the three groups, we employed age-adjusted univariate analysis of covariance (ANCOVA).

A two-sided *p*-value less than or equal to 0.05 was considered to indicate statistical significance. Data analysis was conducted using SPSS V29 (IBM Corp., Armonk, NY, USA).

## 3. Results

### 3.1. Demographic Characteristics

In total, 50 patients with RBD, 51 with OPO and 52 CTRL participated in this study. The RBD group consisted of 17 patients with OI, 17 with HPP and 16 with XLH. The mean age of the OPO group (66.6 ± 10.0) was significantly higher compared to RBD (48.8 ± 15.9) and CTRL (50.8 ± 16.3) (*p* < 0.001), whereas there was no significant difference between RBD and CTRL. All three groups were predominantly female (RBD 26.0% male, OPO 9.8% male, CTRL 26.9% male). The most common educational level was basic across all groups. The employment status differed significantly between the groups (RBD 58.0% employed, OPO 43.1%, CTRL 78.8%, *p* < 0.001). BMI and categories of BMI showed no significant difference between the groups (*p* = 0.16, *p* = 0.20); however, a great portion of subjects were categorized as overweight or obese. Specifically, in the RBD group, 28.0% (*n* = 14) and 24.0% (*n* = 12) were overweight or obese, respectively. The typical number of daily meals ranged from 3 to 4 for all groups and subgroups, though a few individuals in the OPO and CTRL groups reported consuming more than six daily meals (*p* = 0.840). Family status showed no significant differences (*p* = 0.09). Demographic details are presented in Table 1.

### 3.2. Nutritional Categories

**Figure 2:**
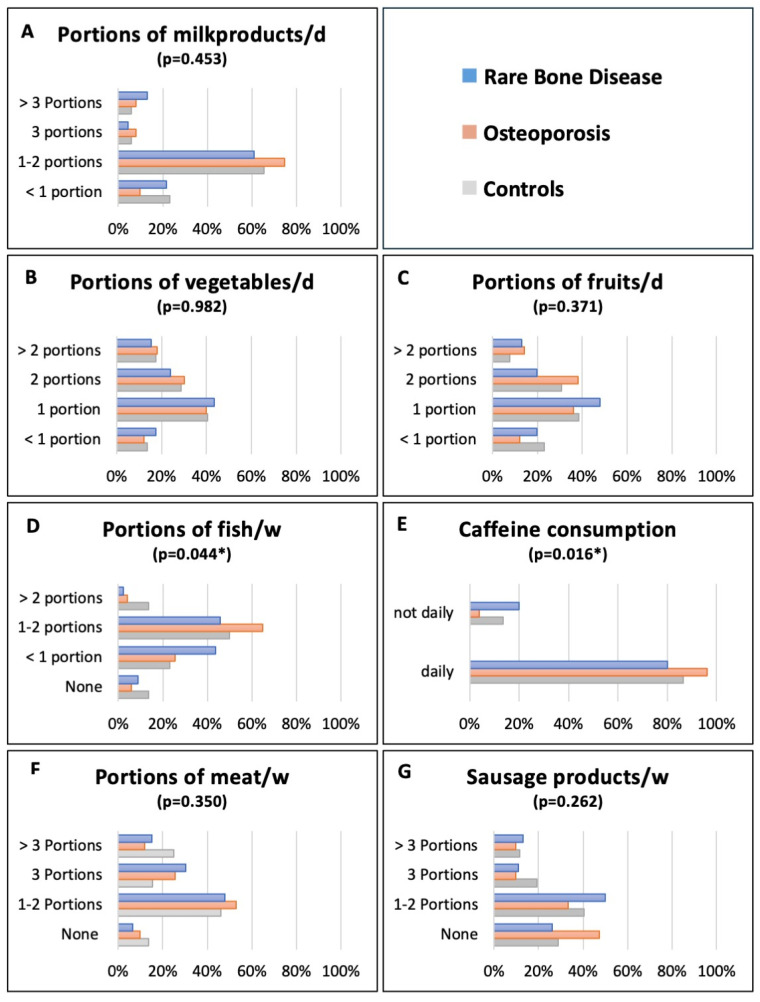
Results of the nutritional domains. Milk and milk product consumption (**A**), vegetable consumption (**B**), fruit consumption (**C**), fish consumption (**D**), caffeine consumption (**E**), meat consumption (**F**) and sausage product consumption (**G**). RBD, rare bone disease; OPO, osteoporosis; CTRL, controls. Significant group differences are marked with an asterisk. W = weekly; d = daily.

The questionnaire consisted of seven nutritional categories: 1. fluid, 2. vegetables and fruits, 3. carbohydrates, 4. milk products, 5. proteins, 6. fat, sweets and oil and 7. snacks. The detailed results of the nutritional questionnaire are reported in Table 2 and presented in Figure 2.

#### 3.2.1. Fluids

Daily fluid intake showed no significant difference between the RBD, OPO and CTRL groups (water/unsweetened drinks—*p* = 0.124, sweetened drinks—*p* = 0.841, light drinks—*p* = 0.699). Daily water or unsweetened drink intake was mainly more than one liter among all groups. Furthermore, the participants mainly drank their hot drinks without sugar, with no significant difference among the groups (*p* = 0.876). Daily caffeine consumption (tea, coffee, energy drinks and soft drinks) showed a significant difference among the three groups (*p* = 0.016), with 96.1% of OPO patients, 80.0% of RBD patients and 86.5% of CTRLs having drinks containing caffeine daily. For fruit/vegetable juice intake, only a minor proportion reported daily consumption; however, the three groups showed a significant difference (*p* = 0.034).

#### 3.2.2. Vegetables and Fruits

Daily vegetable consumption was predominately one portion (equivalent to a handful), followed by two portions among all three groups (*p* = 0.982). In terms of legumes, the majority reported consuming one portion (60–100 g) once a week across the RBD, OPO and CTRL groups (*p* = 0.838). Furthermore, daily fruit intake also showed no significant differences among the groups (*p* = 0.371), with 47.8% of the RBD, 36.0% of the OPO and 38.5% of the CTRL group reporting one daily portion (equivalent to a handful).

#### 3.2.3. Carbohydrates

Starch and cereal products such as bread, rice, or noodles, were primarily consumed daily across all groups, with one to two times a day being the most reported frequency. However, there was no statistically significant difference between the groups in this consumption pattern (*p* = 0.801). In contrast, whole grain products were not consumed on a daily basis by the majority of participants. Nonetheless, there was no significant difference in the consumption of whole grain products among the RBD, OPO and CTRL groups (*p* = 0.203).

#### 3.2.4. Milk Products

Milk and milk products were primarily consumed on a daily basis by each group (*p* = 0.453), with the largest proportion of RBD, OPO and CTRL consuming one to two portions daily. A portion was defined as equivalent to one glass of milk, one cup of yogurt/curd, or one piece of cheese the size of a matchbox. In the OPO group, 90.1% of subjects reported consuming at least one portion daily, compared to 78.1% in the RBD group and 77.0% in the CTRL group.

Additionally, at least three portions daily were reported by 17.3% of RBD, 15.6% of OPO and 11.6% of CTRL. Daily butter or margarine consumption was reported by 66.6% of RBD, 63.2% of OPO and 52.0% of CTRL (*p* = 0.429).

#### 3.2.5. Proteins

One portion (100–120 g) of meat, excluding sausage products, was reported to be consumed at least once weekly by 93.4% of RBD, 90.2% of OPO and 86.6% of CTRL, with the most common frequency being one to two portions weekly (*p* = 0.350). In contrast, 26.1% of RBD, 47.1% of OPO and 28.8% of CTRL participants reported no sausage consumption. Similarly, offal products were predominantly not consumed among all groups.

However, no significant differences were found in the consumption of meat (*p* = 0.350), sausage (*p* = 0.262) or offal products (*p* = 0.349) among the groups. In contrast, weekly fish intake showed a significant difference (*p* = 0.044), with the majority of the subjects consuming one to two portions weekly (45.7% RBD, 64.7% OPO and 50.0% CTRL). Eggs as a source of protein were mainly consumed at a rate of one to two eggs per week (*p* = 0.489).

#### 3.2.6. Fat, Sweets and Oil

The frequency of weekly high-fat meals (e.g., fries, cheese gratinated casserole) significantly differed among the groups (*p* = 0.015). In terms of the consumption of desserts or sweets (e.g., chocolate, cake, pudding), all three groups showed no significant differences (*p* = 0.680). Rapeseed oil was the most commonly used oil for cooking/roasting among all groups, while olive oil was primarily used for non-roasting purposes, such as salad dressings.

#### 3.2.7. Snacks

Salty snack (e.g., chips, salted nuts) consumption showed a significant difference among the groups (*p* = 0.001), in contrast to unsalted nuts or seeds consumption (*p* = 0.168).

### 3.3. Effects of Socioeconomic Status Indicators on the BMI Level

#### 3.3.1. Effect of Educational Level

The ANCOVA assessed the impact of education level and patient type on BMI, adjusted for age. The overall model significantly predicted BMI, F (13, 122) = 2.268, *p* = 0.010, explaining 19.5% of the variance (partial η^2^ = 0.195). Age significantly correlated with BMI, F (1, 122) = 10.438, *p* = 0.002, partial η^2^ = 0.079. Education level approached significance as a predictor, F (2, 122) = 2.481, *p* = 0.088, while patient type did not, F (2, 122) = 0.874, *p* = 0.420. Interaction terms were not significant. Significant pairwise differences were found between patient types, with OPO patients having a lower BMI compared to those with RBD (*p* = 0.014) and CTRLS (*p* = 0.006).

#### 3.3.2. Effect of Marital Status

The inclusion of marital status in the ANCOVA model did not yield significant results, F (17, 117) = 1.486, *p* = 0.11, with a 17.8% explanation of BMI variance by the model (adjusted R^2^ = 0.058). Age remained a significant factor, F (1, 117) = 5.737, *p* = 0.018, partial η^2^ = 0.047. Neither marital status, F (3, 117) = 0.574, *p* = 0.63, nor patient type, F (2, 117) = 0.68, *p* = 0.51, significantly predicted BMI. No significant interactions were found. The effect of patient type on BMI approached significance, F (2, 117) = 2.925, *p* = 0.06, partial η^2^ = 0.048.

#### 3.3.3. Effect of Employment Status

The impact of employment status on BMI was significant, F (9, 128) = 2.305, *p* = 0.02, accounting for about 13.9% of BMI variability (adjusted R^2^ = 0.079). Age again significantly influenced BMI, F (1, 128) = 9.410, *p* = 0.003. Employment status, F (1, 128) = 0.905, *p* = 0.34, and patient type, F (2, 128) = 0.385, *p* = 0.68, did not significantly affect BMI. No significant interactions were observed between patient type and employment status.

Significant differences were observed among patient types; OPO patients had significantly lower BMI compared to RBD and CTRLS (*p* = 0.027 for both comparisons).

## 4. Discussion

In this study, we evaluated the nutritional behavior of patients with rare bone diseases, including XLH, OI and HPP, as well as osteoporosis patients and healthy controls. The questionnaire focused on various nutritional aspects of relevance for bone health and metabolism. Particularly, bone-protective foods were insufficiently consumed in all three study groups.

Calcium plays an important role in bone development and bone health. It was shown that supplementation with calcium or a combination of calcium and vitamin D has beneficial effects on bone mineral density (BMD) and osteoporotic fracture prevention [17]. The primary calcium source should be from the diet [8]. According to the Austrian food recommendations, a daily intake of three portions of milk and milk products is recommended for the healthy population [18]. In the present study, these criteria were not met by most of the subjects in each group. Only a minority of RBD, OPO and CTRL subjects had a daily intake of at least three portions of milk or milk products. Interestingly, especially patients with osteoporosis who are encouraged to meet their daily nutritional calcium needs, have such low numbers. On the other hand, there are also other nutritional sources of calcium, like mineral water or green vegetables, that were not fully evaluated with this questionnaire.

In patients with XLH, calcium intake should be in the normal age-dependent range. However, supplementation with calcium is not recommended in children with XLH as they usually do not have a reduced bone mass or mineral content and because of the potential risk for hypercalciuria [9]. For HPP, there is no evidence for a specific diet, although some suggestions, e.g., for minerals, exist [19]. Often, calcium blood levels are too high in HPP. Consequently, dietary changes/supplementation should be individualized according to medical testing, e.g., laboratory and urine results [19]. For OI, there are recommendations available aiming for a healthy balanced diet. Calcium intake is not necessarily higher for OI compared to the general population. However, as patients with OI are often on antiresorptive agents, calcium and vitamin D levels should be sufficient [20].

As vitamin D controls alimentary calcium absorption, sufficient levels are mandatory. Endogenous production of vitamin D in the skin is dependent on UV-B exposure, explaining seasonal differences in vitamin D levels. However, vitamin D is also available in certain foods like fatty fish (e.g., salmon, mackerel, sardines, cod liver oil), eggs and some types of mushrooms (e.g., shiitake) [21]. In the present study, egg and fish intake was not sufficient to cover the recommended vitamin D requirements. This was also reported by other study groups. The cross-sectional study HELENA (Healthy Lifestyle in Europe by Nutrition in Adolescence) found that vitamin D, folate, iodine and iron intake was about 55% under the recommendations [22]. A recently published study from Finland showed that around 25% did not reach the vitamin D reference value, even though food in Finland is allowed to be fortified with vitamin D [23]. Additionally, the Austrian nutrition report also pointed out that the intake of vitamin D through the diet with the usual foods is not sufficient to meet the reference value for an adequate intake in the absence of endogenous synthesis [24]. Thus, supply via nutrition usually does not cover the needs [25,26], and vitamin D supplementation is needed in patients with bone diseases. The relevance of supplementation is supported by the prevalence of vitamin D deficiency (<50 nmol/L or <20 ng/mL) in Europe, which was estimated at about 40% [27]. 800 IE of cholecalciferol daily is recommended for osteoporosis prevention and as part of osteoporosis treatment [8]. For OI, the daily recommendations vary according to bone-specific treatment and current vitamin D status [20]. In HPP, no specific ranges are given [19]. In XLH, the active form of vitamin D (1,25 vitamin D) is part of the conventional therapy. However, 25 (OH) vitamin D deficiency in children and adults with XLH should be supplemented with native vitamin D [9].

According to current osteoporosis guidelines, protein intake is also relevant for bone health [8]. A systematic review and meta-analysis by Darling et al. revealed that protein intake and BMD or bone mineral content (BMC) had a significant positive correlation, whereas protein intake explained 1–2% of BMD. This was supported by the meta-analysis, which indicated a significant positive influence of protein supplementation on lumbar spine BMD [28]. The Austrian food recommendations recommended at least 1–2 portions of fish per week [18]. These criteria were mainly met by the OPO and CTRL groups. In contrast, less than half of the RBD group met the criteria, mainly due to the low fish intake of XLH patients. Other protein sources are meat, sausage products and eggs. The majority of the subjects in all three groups ate up to three portions of meat per week, as advised by Austrian food recommendations [18]. However, when adding up the weekly consumption of sausage products, a great proportion of all groups exceed the recommended range. The egg intake was mainly in the recommended range of up to three eggs per week.

Both high and low body mass indexes (BMIs) were shown to negatively influence bone health. BMI should be above 20 kg/m^2^ without reaching the overweight/obesity range [8]. In the present study, patients with OI, HPP and XLH, as well as healthy controls were overweight based on their mean BMI. In terms of BMI categories, we further observed that more than half of the patients with RBD, as well as more than one third of the patients with OPO and healthy controls, belong to the categories of overweight and obesity. Johansson et al. showed in their meta-analysis, that the association between fracture risk and BMI is complex. High BMI is a risk factor for upper arm fractures (humerus and elbow). In contrast, low BMI is a risk factor for hip and all osteoporotic fractures but is a protective factor for lower leg fractures. After adjustment of BMD, a high BMI remained a risk factor for upper arm fractures and, additionally, was also a risk factor for all osteoporotic fractures. Furthermore, after BMD adjustment, low BMI persisted as a risk factor for hip fractures but interestingly, was protective for osteoporotic fractures of the tibia, fibula, distal forearm and upper arm [29]. Beside osteoporosis, BMI is also associated with fracture risk in OI. According to Chagas et al., the number of fractures in OI patients was positively correlated with BMI and percentage of body fat and negatively correlated with lean body mass [30]. In adults with XLH, a higher BMI was associated with compromised gait quality by greater lateral trunk lean as well as reduced gait scores [31].

In recent years, bariatric surgery has become a long-term, effective option for weight loss in patients with obesity. Considering the importance of intestinal absorption of micronutrients and vitamins, malabsorptive bariatric surgery appears to be contradictory for patients with bone diseases. Malabsorptive bariatric surgery is associated with an increase in bone turnover and a decrease in BMD, which increases skeletal fragility [32]. In patients after bariatric surgery without mandatory exercise and supplementation of vitamin D, calcium and protein, the decrease in BMD and the change in trabecular bone score were even more pronounced than in patients with supplementation and exercise [33]. As a result, bariatric malabsorptive procedures should be considered with caution in patients with bone diseases, including RBD.

While factors like education, marital status and employment might play significant roles in the general population’s BMI, they do not seem to influence BMI significantly among the different types of patients in our study. We observed that OPO patients generally had a lower BMI, independent of their education, marital status or employment status.

Lastly, caffeine consumption in this study was on a daily basis for the majority of the included subjects. However, caffeine consumption is controversially discussed in the literature. One study showed that a high intake of coffee was associated with a minor reduction in BMD but without increased fracture risk [34]. Another study showed no correlation between coffee consumption and BMD [35].

Our study evaluated the nutritional habits of patients with osteoporosis and rare bone diseases, as well as healthy controls, using a nutritional questionnaire. Despite having a quite large sample size of patients with RBD, certain limitations need to be addressed. Primarily, the used questionnaire was not validated; therefore, as an example, the portion size of different ingredients was not standardized. Furthermore, it is difficult to generalize results from an Austrian cohort, as nutritional habits vary around the world and in different cultures. Moreover, our study did not record the type of fish, mushroom consumption, or sun exposure quantity. It is therefore not possible to draw conclusions about the natural vitamin D intake of our study population. Laboratory parameters, especially vitamin D, would have objective results. However, as we did not evaluate any laboratory parameters, this would be the subject of future studies. Further research is necessary to provide more detailed information on nutritional status, especially on calcium and vitamin D intake in patients with rare bone diseases.

## 5. Conclusions

In conclusion, a major proportion of patients with bone diseases (RBD, OPO) as well as healthy controls were overweight or obese. Furthermore, nearly all included subjects did not meet requirements for milk, milk product and vegetable intake, although a balanced, calcium containing diet is of crucial importance for various bone diseases. Patients with bone diseases should be advised strictly to eat a balanced diet, especially considering their physical limitations. Laboratory examination of vitamin D should be carried out at least at primary consultation. Furthermore, nutritional counseling, the control of BMI and the monitoring of sufficient calcium intake should be considered. Vitamin D does not appear to be sufficiently supplied by the diet, and therefore supplementation should also be considered in patients with bone diseases.

## Figures and Tables

**Table 1:** Basic characteristics of patients’ groups.

Patient Type	RBD (*N* = 50)				OPO (*N* = 51)	CTRL (*N* = 52)	
	OI (*N* = 17)	HPP (*N* = 17)	XLH (*N* = 16)	Total			Group Differences (RBD vs. OPO vs. CTRL)
**Age, mean (SD)**	47.6 (±15.6)	55.9 (±13.9)	42.5 (±16.0)	48.8 (±15.9)	66.6 (±10.0)	50.8 (±16.3)	<0.001
**Gender, male** *N* (%)	5 (29.4)	7 (41.2)	1 (6.3)	13 (26.0)	5 (9.8)	14 (26.9)	0.06
**Family status,** *N* (%)	*	*	*	*	*	*	0.09
Single	4 (23.5)	1 (5.9)	3 (18.8)	8 (16.0)	4 (7.8)	13 (25.0)	
Married or cohabiting	9 (52.9)	10 (58.8)	9 (56.3)	28 (56.0)	25 (49.0)	30 (57.7)	
Divorced	2 (11.8)	3 (17.6)	4 (25.0)	9 (18)	13 (25.5)	5 (9.6)	
Widowed	0 (0.0)	1 (5.9)	0 (0.0)	1 (2.0)	5 (9.8)	4 (7.7)	
**Educational level,** *N* (%)	*	*	*	*	*	*	0.07
Basic	9 (52.9)	7 (41.2)	10 (62.5)	26 (52.0)	19 (37.3)	28 (53.8)	
Secondary	3 (17.6)	2 (11.8)	0 (0.0)	5 (10.0)	16 (31.4)	8 (15.4)	
Tertiary	3 (17.6)	6 (35.3)	6 (37.5)	15 (30.0)	13 (25.5)	16 (30.8)	
**Employment status, employed,** *N* (%)	9 (52.9)	8 (47.1)	12 (75.0)	29 (58.0)	22 (43.1)	41 (78.8)	<0.001
**BMI**, mean (SD)	25.4 (±6.2)	27.2 (±5.1)	25.8 (±5.7)	26.2 (±5.6)	24.2(±3.9)	26.4 (±4.7)	0.16
**BMI category,** *N* (%)	*	*	*	*	*	*	0.20
Underweight (BMI < 18.5)	1 (5.9)	0 (0.0)	0 (0.0)	1 (2.0)	3 (5.9)	1 (1.9)	
Normal (BMI 18.5–24.9)	7 (41.2)	5 (29.4)	7 (43.8)	19 (38.0)	27 (52.9)	20 (38.5)	
Overweight (BMI 25.0–29.9)	4 (23.5)	5 (29.4)	5 (31.3)	14 (28.0)	14 (27.5)	14 (26.9)	
Obese (BMI ≥ 30)	2 (11.8)	6 (35.3)	4 (25.0)	12 (24.0)	3 (5.9)	10 (19.2)	

RBD, rare bone disease; OI, osteogenesis imperfecta; HPP, hypophosphatasia; XLH, X-linked hypophosphatemia; OPO, osteoporosis; CTRL, controls; BMI body mass index. * Missing values: family status—RBD: 4 (8%), OPO: 4 (7.8%), CTRL: 0 (0%); educational level—RBD: 4 (8%), OPO: 3 (5.9%), CTRL: 0 (0%); employment status—RBD: 4 (8.0%), OPO: 1 (2.0%), CTRL: 0 (0%), BMI—RBD: 4 (8%), OPO: 4 (7.8%), CTRL: 7 (13.5%). Data are expressed as a percentage for categorical variables and a mean and ± standard deviation for continuous variables.

**Table 2:** Results of the nutritional questionnaire.

		RBD				OPO	CTRL	Group Differences (RBD vs. OPO vs. CTRL)
		OI	HPP	XLH	Overall			
**Number of daily meals, *N* (%)**	1–2 meals	4 (26.7)	3 (20.0)	3 (18.8)	10 (21.7)	9 (18.0)	9 (17.3)	*p* = 0.840
	3–4 meals	8 (53.3)	12 (80.0)	8 (50.0)	28 (60.9)	34 (68.0)	33 (63.5)	
	5–6 meals	3 (20.0)	0 (0.0)	5 (31.3)	8 (17.4)	6 (12.0)	8 (15.4)	
	6 ≥ meals	0 (0.0)	0 (0.0)	0 (0.0)	0 (0.0)	1 (2.0)	2 (3.8)	
**Water/unsweetened drinks per day, *N* (%)**	≤1 glass (up to 200 mL)	0 (0.0)	1 (6.7)	0 (0.0)	1 (2.2)	0 (0.0)	0 (0.0)	*p* = 0.124
	2 glasses (300–500 mL)	1 (6.7)	0 (0.0)	2 (12.5)	3 (6.5)	3 (6.0)	4 (7.7)	
	3–5 glasses (600–1000 mL)	7 (46.7)	3 (20.0)	4 (25.0)	14 (30.4)	14 (28.0)	5 (9.6)	
	>1 L	7 (46.7)	11 (73.3)	10 (62.5)	28 (60.9)	33 (66.0)	43 (82.7)	
**Sweetened drinks per day, *N* (%)**	≤1 glass (up to 200 mL)	13 (86.7)	13 (86.7)	13 (81.3)	39 (84.8)	42 (84.0)	44 (84.6)	*p* = 0.841
	2 glasses (300–500 mL)	1 (6.7)	1 (6.7)	3 (18.8)	5 (10.9)	7 (14.0)	6 (11.5)	
	3–5 glasses (600–1000 mL)	1 (6.7)	1 (6.7)	0 (0.0)	2 (4.3)	1 (2.0)	1 (1.9)	
	>1 L	0 (0.0)	0 (0.0)	0 (0.0)	0 (0.0)	0 (0.0)	1 (1.9)	
**Light drinks per day, *N* (%)**	≤1 glass (up to 200 mL)	13 (86.7)	15 (100)	15 (93.8)	43 (93.5)	47 (94.0)	50 (96.2)	*p* = 0.699
	2 glasses (300–500 mL)	2 (13.3)	0 (0.0)	1 (6.3)	3 (6.5)	2 (4.0)	1 (1.9)	
	3–5 glasses (600–1000 mL)	0 (0.0)	0 (0.0)	0 (0.0)	0 (0.0)	1 (2.0)	1 (1.9)	
	More than 1 L	0 (0.0)	0 (0.0)	0 (0.0)	0 (0.0)	0 (0.0)	0 (0.0)	
**Sugar per hot drink, *N* (%)**	none	11 (73.3)	12 (80.0)	14 (87.5)	37 (80.4)	43 (86.0)	40 (76.9)	*p* = 0.876
	1 teaspoon	3 (20.0)	2 (13.3)	1 (6.3)	6 (13.0)	6 (12.0)	8 (15.4)	
	2 teaspoons	1 (6.7)	0 (0.0)	1 (6.3)	2 (4.3)	1 (2.0)	3 (5.8)	
	>2 teaspoons	0 (0.0)	1 (6.7)	0 (0.0)	1 (2.2)	0 (0.0)	1 (1.9)	
**Caffeine consumption, *N* (%)**	daily	13 (76.5)	13 (76.5)	14 (87.5)	40 (80.0)	49 (96.1)	45 (86.5)	*p* = 0.016 *
	not daily	4 (23.5)	4 (23.5)	2 (12.5)	10 (20.0)	2 (3.9)	7 (13.5)	
**Fruit/vegetable juice, *N* (%)**	never	2 (13.3)	6 (42.9)	4 (25.0)	12 (26.7)	13 (26.0)	24 (46.2)	*p* = 0.034 *
	not daily	7 (46.7)	4 (28.6)	11 (68.8)	22 (48.9)	30 (60.0)	23 (44.2)	
	1 glass daily (200 mL)	3 (20.0)	3 (21.4)	0 (0.0)	6 (13.3)	6 (12.0)	5 (9.6)	
	>1 glass daily	3 (20.0)	1 (7.1)	1 (6.3)	5 (11.1)	1 (2.0)	0 (0.0)	
**Portions of vegetables per day, *N* (%)**	<1 portion	5 (33.3)	2 (13.3)	1 (6.3)	8 (17.4)	6 (12.0)	7 (13.5)	*p* = 0.982
	1 portion	4 (26.7)	10 (66.7)	6 (37.5)	20 (43.5)	20 (40.0)	21 (40.4)	
	2 portions	3 (20.0)	3 (20.0)	5 (31.3)	11 (23.9)	15 (30.0)	15 (28.8)	
	>2 portions	3 (20.0)	0 (0.0)	4 (25.0)	7 (15.2)	9 (18.0)	9 (17.3)	
**One portion of legumes, *N* (%)**	never	4 (26.7)	4 (28.6)	3 (18.8)	11 (24.4)	9 (18.0)	14 (26.9)	*p* = 0.838
	once a week	8 (53.3)	5 (35.7)	10 (62.5)	23 (51.1)	29 (58.0)	25 (48.1)	
	≥2 times a week	3 (20.0)	5 (35.7)	3 (18.8)	11 (24.4)	12 (24.0)	13 (25.0)	
**Portions of fruits per day, *N* (%)**	<1 portion	5 (33.3)	3 (20.0)	1 (6.3)	9 (19.6)	6 (12.0)	12 (23.1)	*p* = 0.371
	1 portion	4 (26.7)	8 (53.3)	10 (62.5)	22 (47.8)	18 (36.0)	20 (38.5)	
	2 portions	3 (20.0)	3 (20.0)	3 (18.8)	9 (19.6)	19 (38.0)	16 (30.8)	
	>2 portions	3 (20.0)	1 (6.7)	2 (12.5)	6 (13.0)	7 (14.0)	4 (7.7)	
**Starch productions/cereal products per day, *N* (%)**	never/not daily	3 (20.0)	2 (13.3)	2 (12.5)	7 (15.2)	5 (10.0)	4 (7.7)	*p* = 0.801
	1–2 times per day	9 (60.0)	9 (60.0)	11 (68.8)	29 (63.0)	35 (70.0)	36 (69.2)	
	>2 times per day	3 (20.0)	4 (26.7)	3 (18.8)	10 (21.7)	10 (20.0)	12 (23.1)	
**Whole grain products, *N* (%)**	never/once a week	5 (33.3)	9 (60.0)	6 (37.5)	20 (43.5)	16 (31.4)	13 (25.0)	*p* = 0.203
	2–6 times a week	4 (26.7)	3 (20.0)	5 (31.3)	12 (26.1)	11 (21.6)	20 (38.5)	
	once daily	5 (33.3)	2 (13.3)	5 (31.3)	12 (26.1)	20 (39.2)	18 (34.6)	
	several times a day	1 (6.7)	1 (6.7)	0 (0.0)	2 (4.3)	4 (7.8)	1 (1.9)	
**Portions of milk and milk products per day, *N* (%)**	<1 portion	1 (6.7)	5 (33.3)	4 (25.0)	10 (21.7)	5 (9.8)	12 (23.1)	*p* = 0.453
	1–2 portions	10 (66.7)	9 (60.0)	9 (56.3)	28 (60.9)	38 (74.5)	34 (65.4)	
	3 portions	0 (0.0)	0 (0.0)	2 (12.5)	2 (4.3)	4 (7.8)	3 (5.8)	
	>3 portions	4 (26.7)	1 (6.7)	1 (6.3)	6 (13.0)	4 (7.8)	3 (5.8)	
**Eggs per week, *N* (%)**	none	2 (13.3)	5 (33.3)	2 (12.5)	9 (19.6)	6 (11.8)	14 (26.9)	*p* = 0.489
	1–2	6 (40.0)	6 (40.0)	12 (75.0)	24 (52.2)	33 (64.7)	24 (46.2)	
	3	4 (26.7)	3 (20.0)	0 (0.0)	7 (15.2)	5 (9.8)	6 (11.5)	
	>3	3 (20.0)	1 (6.7)	2 (12.5)	6 (13.0)	7 (13.7)	8 (15.4)	
**Portions of meet per week (excl. sausage products), *N* (%)**	none	0 (0.0)	1 (6.7)	2 (12.5)	3 (6.5)	5 (9.8)	7 (13.5)	*p* = 0.350
	1–2 portions	6 (40.0)	7 (46.7)	9 (56.3)	22 (47.8)	27 (52.9)	24 (46.2)	
	3 portions	7 (46.7)	4 (26.7)	3 (18.8)	14 (30.4)	13 (25.5)	8 (15.4)	
	>3 portions	2 (13.3)	3 (20.0)	2 (12.5)	7 (15.2)	6 (11.8)	13 (25.0)	
**Sausage products per week, *N* (%)**	none	3 (20.0)	2 (13.3)	7 (43.8)	12 (26.1)	24 (47.1)	15 (28.8)	*p* = 0.262
	1–2 portions	8 (53.3)	10 (66.7)	5 (31.3)	23 (50.0)	17 (33.3)	21 (40.4)	
	3 portions	1 (6.7)	1 (6.7)	3 (18.8)	5 (10.9)	5 (9.8)	10 (19.2)	
	>3 portions	3 (20.0)	2 (13.3)	1 (6.3)	6 (13.0)	5 (9.8)	6 (11.5)	
**Offal products, *N* (%)**	none	8 (53.3)	8 (53.3)	11 (68.8)	27 (58.7)	34 (66.7)	38 (73.1)	*p* = 0.349
	≤1 portion per month	7 (46.7)	6 (40.0)	5 (31.3)	18 (39.1)	14 (27.5)	11 (21.2)	
	several portions per month	0 (0.0)	1 (6.7)	0 (0.0)	1 (2.2)	3 (5.9)	3 (5.8)	
	1 portion or more per week	0 (0.0)	0 (0.0)	0 (0.0)	0 (0.0)	0 (0.0)	0 (0.9)	
**Portions of fish per week, *N* (%)**	none	1 (6.7)	0 (0.0)	3 (18.8)	4 (8.7)	3 (5.9)	7 (13.5)	*p* = 0.044 *
	<1 portion	5 (33.3)	5 (33.3)	10 (62.5)	20 (43.5)	13 (25.5)	12 (23.1)	
	1–2 portions	9 (60.0)	9 (60.0)	3 (18.8)	21 (45.7)	33 (64.7)	26 (50.0)	
	>2 portions	0 (0.0)	1 (6.7)	0 (0.0)	1 (2.2)	2 (3.9)	7 (13.5)	
**Butter/margarine daily, *N* (%)**	none/not daily	6 (42.9)	6 (40.0)	3 (18.8)	15 (33.3)	18 (36.7)	24 (48.0)	*p* = 0.429
	<2 teaspoons	5 (35.7)	6 (40.0)	3 (18.8)	14 (31.1)	15 (30.6)	8 (16.0)	
	2 teaspoons (=10 g)	2 (14.3)	3 (20.0)	9 (56.3)	14 (31.1)	11 (22.4)	14 (28.0)	
	>2 teaspoons	1 (7.1)	0 (0.0)	1 (6.3)	2 (4.4)	5 (10.2)	4 (8.0)	
**Types of mainly used oil (e.g., salad dressing, not for cooking/roasting), *N* (%)**	olive oil	12 (80.0)	11 (73.3)	14 (87.5)	37 (80.4)	34 (68.0)	39 (78.0)	*p* = 0.208
	rapeseed oil	5 (33.3)	6 (40.0)	7 (43.8)	18 (39.1)	19 (38.0)	11 (22.0)	
	safflower oil	0 (0.0)	0 (0.0)	0 (0.0)	0 (0.0)	2 (4.0)	3 (6.0)	
	corn oil	0 (0.0)	0 (0.0)	3 (18.8)	3 (6.5)	2 (4.0)	4 (8)	
	sunflower oil	1 (6.7)	3 (20.0)	1 (6.3)	5 (10.9)	9 (18.0)	10 (20.0)	
	peanut oil	1 (6.7)	0 (0.0)	0 (0.0)	1 (2.2)	2 (4.0)	2 (4.0)	
	other oils	0 (0.0)	4 (26.7)	0 (0.0)	4 (8.7)	11 (22.0)	15 (30.0)	
**Types of mainly used oil/fat for cooking/roasting, *N* (%)**	olive oil	6 (40.0)	4 (26.7)	7 (43.8)	17 (37.0)	20 (40.0)	17 (34.0)	*p* = 0.098
	rapeseed oil	6 (40.0)	10 (66.7)	8 (50.0)	24 (52.2)	28 (56.0)	26 (52.0)	
	safflower oil	0 (0.0)	1 (6.7)	0 (0.0)	1 (2.2)	1 (2.0)	0 (0.0)	
	corn oil	4 (26.7)	1 (6.7)	6 (37.5)	11 (23.9)	5 (10.0)	6 (12.0)	
	sunflower oil	2 (13.3)	4 (26.7)	4 (25.0)	10 (21.7)	11 (22.0)	21 (42.0)	
	peanut oil	0 (0.0)	0 (0.0)	0 (0.0)	0 (0.0)	2 (4.0)	1 (2.0)	
	margarine	0 (0.0)	2 (13.3)	1 (6.3)	3 (6.5)	2 (4.0)	2 (4.0)	
	coconut fat	0 (0.0)	1 (6.7)	4 (25.0)	5 (10.9)	3 (6.0)	1 (2.0)	
	others	0 (0.0)	0 (0.0)	2 (12.5)	2 (4.3)	5 (10.0)	3 (6.0)	
**Consumption of unsalted nuts or seeds, *N* (%)**	never/rarely	8 (57.1)	12 (80.0)	3 (18.8)	23 (51.1)	25 (52.1)	34 (68.0)	*p* = 0.168
	daily—less than 1 handful	5 (35.7)	2 (13.3)	12 (75.0)	19 (42.2)	16 (33.3)	14 (28.0)	
	daily—1 handful	1 (7.1)	1 (6.7)	1 (6.3)	3 (6.7)	7 (14.6)	2 (4.0)	
	daily—more than 1 handful	0 (0.0)	0 (0.0)	0 (0.0)	0 (0.0)	0 (0.0)	0 (0.0)	
**High-fat meals per week, *N* (%)**	never/rarely	7 (50.0)	6 (40.0)	7 (43.8)	20 (44.4)	33 (68.8)	22 (44.0)	*p* = 0.015 *
	1–2 times	6 (42.9)	8 (53.3)	9 (56.3)	23 (51.1)	15 (31.3)	22 (44.0)	
	≥3 times	1 (7.1)	1 (6.7)	0 (0.0)	2 (4.4)	0 (0.0)	6 (12.0)	
**Consumption of sweets or desserts, *N* (%)**	never/rarely	4 (28.6)	3 (20.0)	6 (37.5)	13 (28.9)	15 (31.3)	12 (24.0)	*p* = 0.680
	1–6 portions per week	7 (50.0)	5 (33.3)	5 (31.3)	17 (37.8)	14 (29.2)	22 (44.0)	
	1 portion per day	3 (21.4)	6 (40.0)	4 (25.0)	13 (28.9)	18 (37.5)	13 (26.0)	
	several portions daily	0 (0.0)	1 (6.7)	1 (6.3)	2 (4.4)	1 (2.1)	3 (6.0)	
**Consumption of salty snacks, *N* (%)**	never/rarely	6 (40.0)	12 (80.0)	9 (56.3)	27 (58.7)	45 (88.2)	26 (51.0)	*p* = 0.001 *
	1–6 portions per week	8 (53.3)	3 (20.0)	6 (37.5)	17 (37.0)	4 (7.8)	23 (45.1)	
	1 portion per day / several portions daily	1 (6.7)	0 (0.0)	1 (6.3)	2 (4.3)	2 (3.9)	2 (3.9)	

RBD, rare bone disease; OI, osteogenesis imperfecta; HPP, hypophosphatasia;, XLH, X-linked hypophosphatemia; OPO, osteoporosis; CTRL, controls. Some respondents marked more than one type of oil; therefore percentages do not round up to 100%. Significant differences were marked with an asterisk.

## Data Availability

The datasets generated and analyzed during the current study are not publicly available due to the risk of indirect identification, where pseudonymized data could inadvertently reveal participants’ identities when cross-referenced with other public information, but they are available from the corresponding author upon reasonable request.

## Supplementary Materials

The following supporting information can be downloaded at: https://www.mdpi.com/article/10.3390/nu16121920/s1, Table S1: Nutritional-Questionnaire.
