# Supplementary material for: Nutritional Behavior of Patients with Bone Diseases: A Cross-Sectional Study from Austria
Source: Nutrients. 2024 Jun 18;16(12):1920. doi: 10.3390/nu16121920 (PMC11206606; doi:10.3390/nu16121920)
Supplement: Supplementary file 1 [file nutrients-16-01920-s001.zip › nutrients-3039844-supplementary.pdf]

Supplementary Table S1: Nutritional-Questionnaire

Name:__________

Date of Birth:_____

Height:__________

Weight:__________

Marital Status:

- o Single
- o Married
- o Cohabiting
- o Divorced
- o Widowed

Highest Completed Education:

- o Primary education
- o High school
- o A-levels
- o University

Are you employed? (Including household management)

- o Yes
- o No

1. How many meals do you eat per day?
   Breakfast, lunch, dinner, snacks - please consider even a piece of fruit or a yogurt counts as a snack.
   - o 1-2 meals
   - o 2-3 meals
   - o 5-6 meals
   - o More than 6 meals

2. How much water and/or unsweetened drinks such as fruit or herbal teas do you drink per day?
   - o None or less than one glass (200ml)
   - o 2 glasses (300-500ml)

- 3-5 glasses (600-1000ml)
- More than 1 liter

3. How much sweetened drinks do you drink per day? Examples: soft drinks, lemonades, iced tea, chocolate drinks, energy drinks
    - None or less than one glass (200ml)
    - 2 glasses (300-500ml)
    - 3-5 glasses (600-1000ml)
    - More than 1 liter

4. How many light drinks do you consume per day?
    - None or less than 1 glass (200ml)
    - 2 glasses (300-500ml)
    - 3-5 glasses (600-1000ml)
    - More than 1 liter

5. Indicate the number of cups/glasses of caffeinated beverages you drink daily.

    Coffee (125ml)_________

    Cappuccino (125ml)_____

    Espresso (50ml)________

    Green Tea (125ml)______

    Black Tea (125ml)_______

    Cola Drinks (300ml)______

    Energy Drinks (250ml)____

6. How much sugar do you usually add to your hot beverages?
    - None
    - One teaspoon
    - Two teaspoons
    - More than two teaspoons

7. Do you drink fruit or vegetable juices?
    - Never
    - Not daily

- 1 glass daily (200ml)
- Several times daily (more than 200ml)

8. How many portions of vegetables do you eat per day?
   Examples: 1 portion (1 handful) equals 1 vegetable side dish, 2 carrots, 1 mixed salad
   - None or less than one portion
   - 1 portion
   - 2 portions
   - More than 2 portions

9. How often do you eat 1 portion of legumes?
   Examples: 1 portion equals 60-100g (raw weight) of peas or lentils.
   - Never
   - Once a week
   - At least twice a week

10. How many portions of fruit do you eat per day?
    Examples: 1 portion (1 handful) equals 1 medium apple, 1 banana.
    - None or less than 1 portion
    - 1 portion
    - 2 portions
    - More than 2 portions

11. How often do you eat starch or cereal products per day?
    Examples: bread, rice, pasta, potatoes.
    - Never or not daily
    - 1-2 times a day
    - More than 2 times a day

12. How often do you eat whole grain products?
    Examples: whole grain bread, whole grain rice, whole grain pasta, cereal flakes like oats or barley.
    - Never or once a week

- o 2-6 times a week
- o Once a day o Several times a day
- o Several times daily

13. How many portions of milk and milk products do you consume per day?
    Examples: 1 portion equals 1 glass of milk, 1 cup of yogurt, 1 cup of curd or 1 piece of cheese in the size of a matchbox.
    - o None or less than 1 portion
    - o 1-2 portions
    - o 3 portions
    - o More than 3 portions

14. How many eggs do you eat per week?
    - o None
    - o 1-2
    - o 3
    - o More than 3 eggs

15. How many portions of meat or poultry (excluding sausage products) do you eat per week?
    Examples: 1 portion (100-120g) equals 1 chicken drumstick.
    - o I do not eat meat or poultry.
    - o 1-2 portions
    - o 3 portions
    - o More than 3 portions

16. How often do you eat sausage products per week?
    Examples: 1 portion equals 1 sausage, 5-8 slices of salami, or 2-4 slices of cold cuts.
    - o I don't eat any sausage products.
    - o 1-2 portions
    - o 3 portions
    - o More than 3 portions

17. How often do you eat offal products?

    Examples: 1 portion (120g) equals 1 piece of liver, kidney, brain.

    - I don't eat any offal products.
    - Less than 1 portion per month / 1 portion per month
    - Several portions per month
    - 1 portion per week or more

18. How many portions of fish do you eat per week?

    Examples: 1 portion (100-120g) equals 1 medium fish fillet or 4-5 fish sticks.

    - I don't eat any fish.
    - Less than 1 portion
    - 1-2 portions
    - More than 2 portions

19. How much butter/margarine do you use daily?
    - None or not daily
    - Less than 2 teaspoons
    - 2 teaspoons
    - More than 2 teaspoons

20. Which oil do you mainly use in cold dishes, e.g., for preparing salad dressing?
    - Olive oil
    - Rapeseed oil
    - Safflower oil
    - Corn oil
    - Sunflower oil
    - Peanut oil
    - Other

21. Which oil or fat do you mainly use in hot dishes, e.g., for cooking/roasting?
    - Olive oil
    - Rapeseed oil
    - Safflower oil
    - Flaxseed oil

- Corn oil
- Sunflower oil
- Peanut oil
- Margarine
- Coconut oil
- Other

22. How often do you eat unsalted nuts or seeds?

    Examples: almonds, peanuts, hazelnuts, pumpkin seeds, flaxseeds.
    - Never or rarely
    - Daily – less than a handful
    - Daily – a handful
    - Daily – more than a handful

23. How often do you eat high-fat meals per week?

    Examples: breaded schnitzel, French fries, casserole baked with cheese.
    - Never or rarely
    - 1-2 times
    - At least 3 times

24. How often do you eat sweets or desserts?

    Examples: 1 portion equals 2 bars of chocolate, 1 piece of cake, 1 pudding.
    - Never or rarely
    - 1-6 portions per week
    - 1 portion per day
    - Several portions daily

25. How often do you eat salty snacks?

    Examples: 1 portion equals 2 handfuls of chips, pretzels or 1 handful of salted nuts.
    - Never or rarely
    - 1-6 portions per week
    - 1 portion per day or several portions daily

The questionnaire was originally in German language and translated into Englisch.
